# Thank you to reviewers

**DOI:** 10.1093/eurpub/ckad004

**Published:** 2023-02-03

**Authors:** Peter Allebeck, Diana Delnoij, Alastair Leyland, Walter Ricciardi, Róza Ádány, Stefania Boccia

A scientific journal exists only because of the good will and assistance of its reviewers. We can only publish approximately 25% of the papers we receive. In order to publish really high-quality papers, we depend on the expertise of many people. In 2022, a total of 344 reviewers provided their expert opinions for us to decide about the papers that we should publish. Their names are listed below and we would like to take this opportunity to thank them.

Muhammad Abbas, China

Balázs Ádám, Hungary

Róza Ádány, Hungary

Mathilde Adsersen, Denmark

Gunnar Ågren, Sweden

Charles Agyemang, Netherlands

Johan Åhlen, Sweden

Abdulbaghi Ahmad, Sweden

Adebisi Akande, Canada

Yannis Alamanos, Greece

Francois Alla, France

Sara Allin, Canada

Javier Almela-Baeza, Spain

Ridwanul Amin, Sweden

Peter Angelopoulos, Greece

Johanna Anneser, United States

Cigdem Apaydin Kaya, Turkey

Ramiz Arabaci, Turkey

Benjamin Aretz, Germany

David Armstrong, United Kingdom

LUCIA ARTAZCOZ, Spain

Barbara Artnik, Slovenia

Peter Aspinall, United Kingdom

Pilar Astier, Spain

Bontha Babu, India

Sekene Badiaga, France

Birna Baldursdottir, Iceland

Angelo Barbato, Italy

MArtina Barchitta, United States

Pepita Barlow, United Kingdom

Orna Baron-Epel, Israel

Ben Barr, United Kingdom

Xavier Bartoll, Spain

Sarah Basharat, Pakistan

Linda Bell, United Kingdom

Johannes Beller, Germany

Netta Bentur, Israel

Dániel Bereczki, Hungary

Lisa Berkman, United States

Marriot Bernadette, United States

Vesna Bjegovic Mikanovic, Serbia

Jonas Björk, Sweden

Anders Bjorkman, Sweden

Jenni Blomgren, Finland

Edwin Boezeman, Netherlands

Henrik Bøggild, Denmark

Michaël Boissonneault, Netherlands

Alexander Bor, Denmark

Guilherme Borges, Mexico

Alberto Borgetti, United States

Jennifer Boyd, United Kingdom

Erica Briones-Vozmediano, Spain

Óscar Brito Fernandes, Netherlands

Corinne Brooks, United States

Heather Brown, United Kingdom

Laura Brunelli, Italy

Anna Bucsics, Austria

Alex Burdorf, Netherlands

Andrea Buron, Spain

Hermann Burr, Germany

Bo Burström, Sweden

Marcel Buster, Netherlands

Noriko Cable, United Kingdom

Chiara Cadeddu, Italy

Gabriella Cadoni, Italy

Timothy Callaghan, United States

Cristina Canova, Italy

Simon Capewell, United Kingdom

Martin Caraher, United Kingdom

Margarida Cardoso, Portugal

S Carol, United States

Ewan Carr, United Kingdom

Susan Carr, United Kingdom

Pilar Carrasco-Garrido, Spain

R Catalano, United States

Angela Chang, Denmark

Boris Chapoton, France

Frank Chesser, United States

Jenny Cisneros, Sweden

Sheelah Connolly, Ireland

Elisabeth Corker, United States

Claudia Costa, Portugal

Hannah Covert, United States

Matty Crone, Netherlands

Paolo Crosignani, Italy

Carol B. Cunradi, United States

Sarah Cuschieri, Malta

Shaonong Dang, China

Zuzana Dankulincova, Slovakia

Mike Daube, Australia

Sascha de Breij, Netherlands

Pauline de Heer, Netherlands

Eline de Heus, Netherlands

Judith de Jong, Netherlands

Evelyne De Leeuw, Australia

alessandro de matteis, United Kingdom

Augusto Cesar De Moraes, Brazil

Corrado De Vito, Italy

DJH Deeg, Netherlands

Patricia M. Dekkers-Sánchez, Netherlands

Stefaan Demarest, Belgium

Evangelia Demou, United Kingdom

alexis descatha, France

Finn Diderichsen, Denmark

Henriëtte Dijkshoorn, Netherlands

Charalabos-Markos Dintsios, Germany

Kritika Dixit, Nepal

Annette J Dobson, Australia

Felıcitas Domınguez-Berjon, Spain

Dunja Dreesens, Netherlands

Ruth Dundas, United Kingdom

Xavier Duran, Spain

Anna Dzielska, Poland

Frida Eek, Sweden

Mikael Ekblad, Finland

Ola Ekholm, Denmark

Anna Mia Ekström, Sweden

George Ellison, United Kingdom

Leonardo Emberti Gialloreti, Italy

Eric Emerson, United Kingdom

Erdem Erkoyun, Turkey

Miriam Evensen, Norway

Michelle Falkenbach, United States

Daniel Falkstedt, Sweden

Sara Farina, Italy

Michael Farrell, Australia

Albert Faye, France

Ariadna Feliu, Spain

Carmine Finelli, Italy

Florian Fischer, Germany

Pär Flodin, Sweden

Carla Fornari, Italy

Stefan Fors, Sweden

Thomas Fröhlich, Germany

Sari Fröjd, Finland

karine gallopel-morvan, France

Ana Gama, Portugal

Thomas Gamsjäger, Austria

Heike Garritsen, Netherlands

Vijay Gc, United Kingdom

Laurent Gerbaud, France

Mariette Gerber, France

Ali Ghaddar, Spain

Delaram Ghodsi, Iran

MONICA GIANCOTTI, Italy

Katja Gillander Gadin, Sweden

Alejandro Gil-Salmerón, United Kingdom

Linn Gjersing, Norway

Pere Godoy, Spain

Rosa Gofin, Israel

Unni Gopinathan, Norway

Scott Greer, United States

Aikaterini Grimani, United States

Maria Gualano, Italy

C Guijarro, United States

Luc Hagenaars , United States

Faisal Hakeem, United Kingdom

Christian Hakulinen, Finland

Torleif Halkjelsvik, Norway

Samah Hayek, Israel

K Hedna, Sweden

N Heinonen, United States

Jolyn Hersch, Australia

Anders Hjern, Sweden

Theodore Holford, United States

Zakir Hossin, Sweden

H Hoven, Germany

Kuo-Cherh Huang, Taiwan

D Ivankovic, United States

Anant Jani, United Kingdom

Staffan Janson, Sweden

Leticia Januario, Sweden

Bengt Järvholm, Sweden

Domantas Jasilionis, Germany

Dmitri Jdanov, Germany

Junia Joffer, United States

T Jörgensen, Denmark

Karsten Jørgensen, Denmark

Leena Kaila-Kangas, Finland

Michelle Kelly Irving, France

Carolin Kilian, Germany

Kennedy Kipkoech, United States

Marte Kjøllesdal, Norway

Cecile Knai, United Kingdom

AK Knudsen, Norway

Sari Kovats, United Kingdom

Slawomir Koziel, Poland

Alfgeir Kristjansson, Iceland

Anton Kunst, Netherlands

Sandra Kuntsche, Australia

Daniel La Parra-Casado, Spain

Carlo La Vecchia, Italy

Mikko Laaksonen, Finland

Ulrich Laaser, Germany

Angela Labberton, Norway

Maude Laberge, Canada

Tea Lallukka, Finland

Paul Lambert, United States

Anthony D. LaMontagne, Australia

Chris Lane, United States

Peter Lansberg, Netherlands

Seo Yoon Lee, Korea (the Republic of)

Dan Lewer, United Kingdom

Alastair Leyland, United Kingdom

Giovanna Liuzzo, United States

Francesco Longo, Italy

E Lothian, United Kingdom

Marty Lynch, United Kingdom

Ronan Lyons, United Kingdom

Georgina MacArthur, United Kingdom

Anne MacFarlane, Ireland

Anna Macios, United States

João Paulo Magalhães, Portugal

Manfred Maier, Austria

Steven Mann, United Kingdom

Tanja Marschall, Germany

Joachim Marti, Switzerland

Leslie Martin, United States

Jolanda Mathijssen, Netherlands

Donovan Maust, United States

Laia Maynou, United Kingdom

Joanna Mazur, Poland

Mary McEniry, United States

Phil McHale, United Kingdom

Martin McKee, United Kingdom

Courtney McNamara, Norway

Emmanouil Mentzakis, United Kingdom

Julien Mercille, United States

Ray Merrill, United States

Anu Molarius, Sweden

Linda Montanari, Portugal

Maria Mourão-Carvalhal, Portugal

Massimo Mucciardi, Italy

Csilla Nagy, Hungary

Neetu S. Neetu Abad Abad, United States

Davide Negrini, Italy

Bertalan Németh, Hungary

Melanie Nichols, Australia

Suzanne Nielsen, United States

Peter Nilsson, Sweden

Christiana Nöstlinger, Belgium

Mario Cesare Nurchis, Italy

S Ofori, United States

Leslie Ogilvie, Germany

Orkan Okan, Germany

Hans Ossebaard, Netherlands

Fred Paccaud, Switzerland

Antoine Pariente, France

Sam Parsons, United States

Domenico Pascucci, Italy

Julian Perelman, Portugal

Pamela Pereyra-Zamora, Spain

VLADIMIR PETROVIC, Serbia

Isabelle Peytremann-Bridevaux, United States

Angelo Maria Pezzullo, Italy

H Susan J Picavet, Netherlands

Sylvain Pichetti, France

Peter Piko, Hungary

Tom Platteau, Belgium

Thomas Plümper, Austria

Andrea Poscia, Italy

Valentina Prevolnik Rupel, Slovenia

Tina PURNAT, Sweden

Pekka Puska, Finland

Xun Qi, United States

Justina Račaitė, Lithuania

Cecilia Radkiewicz, United States

Ossi Rahkonen, Finland

Syed Rahman, Sweden

Francesco Ramponi, Spain

Sofia Ravara, Portugal

Sarah Reed, United Kingdom

Sijmen Reijneveld, Netherlands

Rainer Reile, Estonia

Grégoire Rey, France

Sofia Ribeiro, Portugal

Vladimir Rogalewicz, Czech Republic

Hana Ross, South Africa

Annalisa Rosso, Italy

Ameed Saabneh, Israel

Henrique Sachse-Bonhof, Netherlands

Muhammad Saeed, Pakistan

Laura Samuel, United States

Miguel San Sebastian, Sweden

Fabian Sanchis-Gomar, Spain

P Santia, United States

Cornelia Santoso, Hungary

Ingrid Seinen, Netherlands

Mehmet Sukru Sever, Turkey

Mohsen Shamsi, Iran (the Islamic Republic of)

Wensong Shen, Hong Kong

Koichiro Shiba, United States

Ilana Shoham-Vardi, Israel

Colin Shore, United Kingdom

Karri Silventoinen, Finland

Florian Slimano, France

Maja Sočan, Slovenia

Lotti Sofia, Italy

Jeppe Karl Sørensen, Denmark

Emily South, United Kingdom

Gordana Stankovska, United States

Kaspar Staub, Switzerland

Agnes Stenius-Ayoade, Finland

Tom Sterud, Norway

Abigail Stevely, United Kingdom

Susanne Stolpe, Germany

Jack Stone, United States

Stefan Storcksdieck genannt Bonsmann, Germany

Cosmo Strozza, Denmark

Jukka Takala, Finland

Dudley Tarlton, United States

Nicholas Taylor, Australia

Perihan Torun, Turkey

Swenne G van den Heuvel, Netherlands

J. van der Velden, Netherlands

Hans van der Wouden, Netherlands

Christel van Dijk, Netherlands

Carla van El, Netherlands

Marieke van Hoffen, Netherlands

Geert van Kemenade, Netherlands

Jeroen van Meijgaard, United States

Hanna van Solinge, Netherlands

Maarten van Wijhe, Denmark

Ko van Wouwe, Netherlands

Sander van Zon, Netherlands

Carine Vande Voorde, Belgium

Leonardo Villani, Italy

Michal Vinker-Shuster, United States

Pekka Virtanen, Finland

Sven Voigtländer, Germany

David Walsh, United Kingdom

Heather Wardle, United States

Roger Webb, United Kingdom

Dominic Weinberg, Netherlands

Dr. H. Gilbert Welch, United States

Shicheng Yu, China

Virginia Zarulli, Denmark

Mateusz Zatonski, United Kingdom

Jennifer Zeitlin, France

Caixia Zhang, China

Pengxiang Zhao, Sweden

Cheryl Zlotnick, Israel

Irini Zografaki, Greece

Corina-Aurelia Zugravu, Romania

